# Strontium Bromide in Epilepsy

**Published:** 1893-09

**Authors:** 


STRONTIUM BROMIDE IN EPILEPSY.—The constantly increasing number of incurable epileptics, both in asylum and at large, occasions an ever-growing demand for new drugs, from which we may at least hope to effect some improvement, in either their physical condition, or a diminution of the number of seizures.

Among the recent applicants for medical favor in this line has been the bromide of strontium (Paraf-Javal), purporting to be a salt free from the impurities of the ordinary commercial article, which render it unfit for continued use, or even poisonous in moderate doses. This statement as to its non-toxic action we have found to be well founded, no evil result having followed 30-grain doses repeated thrice daily, and no case that has been treated with the salt has shown other than beneficial results. Above all, we have to note continued absence of a bromide acne (even the disappearance of the rash, though it was present when the use of the strontium was commenced), a very much lessened somnolent effect, the patients without exception appearing brighter and more cheerful under its use than with the sodium salt, and finally certain excitable cases were less quarrelsome after a seizure, than under the every-day treatment; point all of very considerable value, both in private and asylum practice.—*Times and Register*, July 1893.

---

PAPOID.—Here was a case of big clot in the stomach of a drunkard whose gastric juice failed to have any impression upon, and which violent efforts at emesis failed to remove, but which papoid dissolved quickly, thereby saving the man's life.

In many cases of indigestion it is a difficult matter to make a diagnosis as to whether the trouble is in the stomach or in the small intestine. If in the intestine, and pepsin is given, no good results. If the trouble is located in the stomach, and pancreatine is given, the same condition pertains, but in each case papoid is given, the result is a solution quickly of the whole trouble.

I have used it largely in the various forms of dyspepsia and indigestion with most excellent results—*N. Eng. Med. Monthly.*

"Coca" has maintained its reputation as a powerful nerve stimulant, being used with good results in nervous debility, opium and alcohol habit, etc. The highly variable character of the commercial drug makes it uncertain however. Robinson's Wine Coca (see ad.) we believe to be a uniformiy active article, it being prepared from assayed leaves, the percentage of Cocaine being always determined by careful assay.

